# Older People and Unpaid Carers’ Experiences of Hospital-at-Home

**DOI:** 10.5334/ijic.10181

**Published:** 2025-12-04

**Authors:** Mark Tompkins, Robin Miller, Denise Tanner

**Affiliations:** 1 University of Birmingham, United Kingdom

**Keywords:** Hospital-at-Home, older people, unpaid carers, integrated care, qualitative research

## Abstract

Hospital-at-Home (HaH) delivers hospital-level treatment in the home, whether people’s own dwelling or a care home. Its intention is to replicate medical interventions available in hospitals in familiar and less distressing surroundings for older people and better coordinate care around the needs of the individuals and their unpaid carers. This study set out to hear directly from those most closely involved, older people themselves, the unpaid carers who support them, and the professionals delivering the service. Drawing on forty-three in-depth interviews, the research highlights both advantages and tensions within the model. Many participants described HaH as respectful, personal, and more attentive than hospital care. Carers welcomed the speed and attentiveness of the service but often found their responsibilities increased, sometimes to a challenging degree. Professionals valued the opportunity to work in a more person-centred way while also pointing to practical obstacles around risk, resources, and coordination with wider services.

## Introduction

Globally, the population is ageing at an unprecedented rate, so by 2050, one in five people will be over 60 [1]. In the UK, the pressures on health and social care are long-standing, sharpened by COVID-19, rising demand, and workforce shortages, with concerns about how best to respond to an ageing population in which increasing numbers of people have multiple and long-term conditions [2,3]. For older people themselves, repeated hospital admissions can bring new risks, such as infection, delirium, or loss of independence, yet delays in social care assessments often leave people without adequate support at home [4,5].

Against this backdrop, alternative models of integrated care have gained attention. One such approach is HaH, which combines a multi-disciplinary team and remote technologies to provide acute-level interventions at home. HaH services can be deployed to prevent a hospital admission or support earlier discharge, and by reducing exposure to an inpatient setting, can mitigate harms linked to inpatient care while improving comfort and satisfaction [6].

The HaH service studied in this research operates as a commissioned element of standard NHS provision within an urban Integrated Care System, rather than a short-term pilot. Referrals are made primarily by hospital clinicians to support early discharge, although general practitioners and community services can also initiate admission avoidance referrals. The service is funded through NHS commissioning arrangements and delivered by a multidisciplinary team comprising doctors, nurses, therapists, and pharmacists employed directly by the NHS Trust responsible for the local acute hospital. Core features include daily medical oversight, access to home-based diagnostics (such as point-of-care testing and portable imaging), and coordination with social care to ensure safe discharge and continuity. This structure reflects national guidance on HaH as an alternative to inpatient admission, integrating acute and community care around the individual [6,7].

Most research to date on HaH has centred on safety and costs, with far less attention paid to the lived experiences of older people and their unpaid carers and to the collaborative processes required to deliver this model of care successfully. This study set out to fill that gap by gaining the perspectives of older people, carers, and professionals in a HaH service within an urban setting.

## Research aims

- To investigate experiences of older people, unpaid carers, and professionals.
- To examine how HaH interacts with wider health and social care services.
- To draw lessons for future policy and practice.

## Methodology

The study adopted a qualitative, phenomenological approach to capture the lived experiences of those involved. Forty-three people participated: eleven older people who had received HaH, eleven unpaid carers, and twenty-one professionals. Purposeful sampling ensured direct, recent experience. Interviews were semi-structured, lasting up to an hour. Older people and carers were interviewed face-to-face, and professionals were interviewed online via Microsoft Teams.

Data was analysed using reflexive thematic analysis [8], with the support of NVivo software. Themes were generated inductively, shaped by participants’ accounts rather than imposed at the outset. Ethical approval was secured (RG_21-005), and informed consent was obtained from all participants. In accordance with the Mental Capacity Act, individuals with marked cognitive impairments were not recruited, as valid consent could not be assured within the scope of this study. The researcher’s professional background as a hospital social worker helped build rapport and informed a reflexive approach to analysis, with transparency maintained concerning positionality.

The study does, however, have its limitations. It was carried out within a single region of the UK, and the findings cannot automatically be assumed to reflect experiences in areas where HaH services are structured or delivered differently. In addition, participation was voluntary, raising the possibility of self-selection bias, whereby those with particularly strong or clear views may have been more inclined to contribute.

## Findings

### Experiences of Older People

Older people consistently described the clinical care received at home as safe and reliable, with strong confidence in staff competence. What they emphasised just as much was the time taken by professionals to explain treatment and listen. This contrasted with hospital encounters, which often felt hurried or impersonal. At home, attention was focused solely on them, not dispersed across a ward, contributing to dignity and respect. Familiar surroundings and proximity to family reinforced comfort and a sense of independence.

### Experiences of Unpaid Carers

For unpaid carers, the picture was mixed. Many had not heard of HaH until a crisis arose, typically via a GP or ambulance referral, reflecting low public visibility. Carers valued the reduced stress of avoiding the hospital, but responsibilities often increased, affecting employment and sometimes their own health. Even so, they praised the thoroughness of HaH and the open communication with both themselves and the older person. Coordination, however, was uneven. While HaH teams were well organised internally, links with GPs and social care could be patchy, leaving gaps and extra strain for unpaid carers.

### Experiences of Professionals

Professionals described adapting to a relatively new way of working. Managing clinical risk was a recurring concern. Unlike in a hospital, responsibility often rested with one individual, who had to balance patient choice with safety. Escalating issues outside normal hours could be difficult, leaving some feeling exposed. Within HaH teams themselves, however, inter-professional working was often described positively. Nurses, doctors and therapists highlighted the value of drawing on one another’s expertise, with regular handovers and day-to-day communication helping to build trust and a shared sense of purpose. This contrasted with the challenges that emerged at the boundaries with GPs and social care, where communication gaps could at times lead to avoidable admissions.

Care delivered in the home was viewed as more conducive to person-centred practice, enabling holistic assessment and family involvement. However, carers’ own needs were rarely acknowledged formally. Practical difficulties remained, including patchy training, equipment that was not always available, and occasional uncertainty around role boundaries. More broadly, professionals felt that weak integration with local authority services remained the most problematic issue.

## Implications for Integrated Care and Future Research

Findings suggest HaH as a promising step towards more integrated and person-centred care. Older people valued being treated in their own surroundings and the dignity this afforded. Carers appreciated being included in discussions, though often at personal cost. Professionals recognised the strengths of the approach but were equally frank about implementation challenges, citing resource shortages, risk management, and weak inter-agency coordination.

## Key Implications

- **Equity and access**: HaH currently works best for those with carer support. Future models must include people living alone and with cognitive impairments.
- **Integration**: stronger coordination with local authority social care is essential to ensure safe discharge and continuity. Joint or co-located teams may help.
- **Support for carers**: routine carer assessments and structured support are vital, in line with statutory duties under the Care Act 2014.
- **Training and workforce**: staff need preparation in shared decision-making, inter-agency working, and holistic care, not only in clinical tools.
- **Public awareness**: campaigns and GP engagement could reframe HaH as a planned alternative, not simply an emergency response.
- **Future research**: should explore equity of access, long-term sustainability, and experiences of more diverse groups.

## Conclusion

HaH represents a promising development in acute care for older people. This study shows that it is experienced by many as safer, more personal, and more respectful than a hospital. However, the model also brings challenges: increased responsibilities for carers, complex decisions for professionals, and persistent integration difficulties with social care. If these issues are addressed, HaH has the potential to move beyond being a policy aspiration to become a fully embedded part of integrated care for older people.
